# Microscopically tuning the graphene oxide framework for membrane separations: a review

**DOI:** 10.1039/d1na00483b

**Published:** 2021-08-10

**Authors:** Bofan Li, Chen-Gang Wang, Nayli Erdeanna Surat'man, Xian Jun Loh, Zibiao Li

**Affiliations:** Institute of Materials Research and Engineering, Agency for Science, Technology and Research (A*STAR) 2 Fusionopolis Way, Innovis #08-03 Singapore 138634 li_bofan@imre.a-star.edu.sg lizb@imre.a-star.edu.sg; Department of Materials Science and Engineering, National University of Singapore Singapore 117574 Singapore

## Abstract

Membrane-based separations have been widely applied in gas, water and organic solvent purifications to reduce energy consumption and minimize environmental pollution. In recent years, graphene oxide (GO) membranes have attracted increasing attention due to their self-assembly ability and excellent stability. In this review, publications within the last 3 years on microscopically tuning the GO framework are summarized and reviewed. Various materials, including organic molecules, polymers, inorganic particles, ions and 2D materials, have been deployed to intercalate with GO nanosheets. Due to the varied interlayer spacing and packing structure, the developed GO composites exhibit enhanced stabilities and separation performances. In addition, designing horizontal GO membranes and functionalizing GO nanosheets have also been reported to improve the performance. This review sheds light on the techniques to microscopically tune the GO framework and the resulting macroscopic changes in membrane properties and performances.

## Abstract 2

This mini-review summarizes the publications within the last 3 years on microscopically tuning the interlayer spacing, lateral size and functional group to improve the properties and separation performance of graphene oxide membranes.

## Graphical abstract

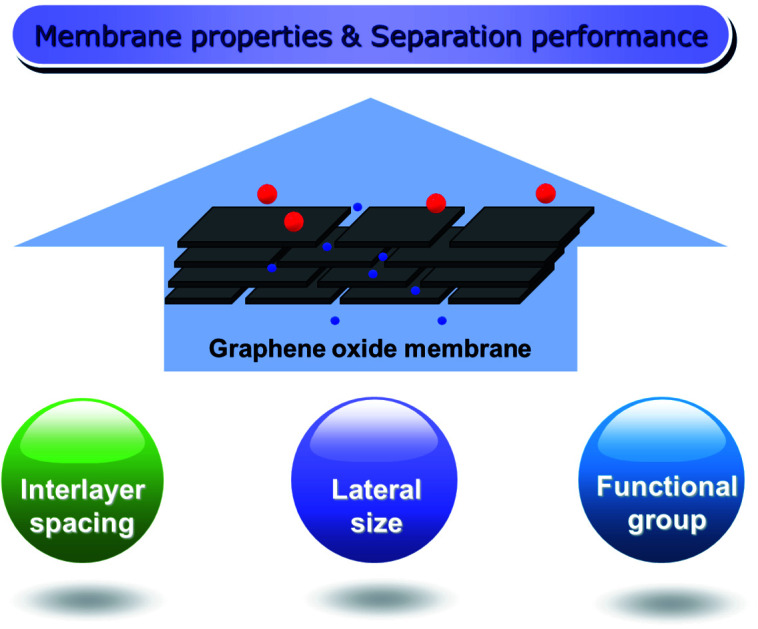

## 1. Introduction

**Fig. 1:**
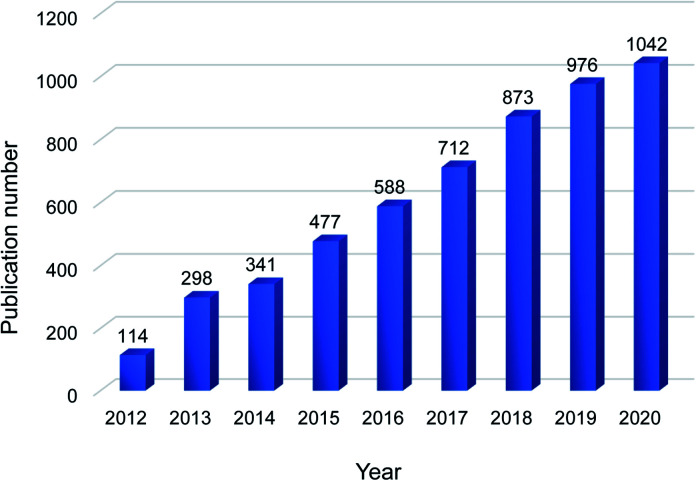
Number of publications on GO membranes in the past 10 years. The data were obtained on 27^th^ May 2021 from Scopus by searching “graphene oxide” and “membrane” in the article title, abstract and keywords.

Chemical separations are applied in almost every production process and account for 10–15% of the world's energy consumption.^1,2^ Thermal separation processes, such as distillation and evaporation, are commonly employed but they are energy intensive. Membrane-based separations are emerging as sustainable separations as they can use 90% less energy than distillation and emit less CO_2_.^1^ Nowadays, various materials have been applied for membrane development, including polymers, ceramics and carbon-based materials.^3–6^ Carbon-based materials, like carbon nanotubes, graphene and its derivative graphene oxide (GO), are exceptional in their electrical, mechanical and thermal properties.^7^ Among them, GO has attracted a lot of interests in the last decade due to its excellent dispersibility and self-assembly ability, with an increasing number of publications on GO-based membranes year by year (Fig. 1).

GO consists of a single layer of carbon atoms arranged in a honeycomb lattice with oxygen containing functional groups on the edges and basal planes.^8^ As GO contains abundant oxygen functional groups, it can be well dispersed in some polar solvents, such as water, *N*-methyl-2-pyrrolidone and ethyl glycol.^9^ This enables GO to be easily processed and assembled into a laminar structure to function as a membrane. There are many approaches to assemble GO membranes (GOMs), for example, pressure-assisted filtration,^10,11^ layer-by-layer deposition,^12–14^ drop-casting,^15^ spin coating^16^ and spray coating.^17^ Different assembling methods could result in different packing structures and separation performances.^18^ Among them, pressure-assisted filtration is widely employed due to its ease of operation. The GO dispersion is forced to pass through a membrane substrate *via* pressurization or applying vacuum. The GO nanosheets are thus stacked on the membrane substrate to form a laminar framework.^18^ In this process, the rate of filtration and the concentration of GO dispersion can affect the structure and performance of GOMs.^19^ Layer-by-layer deposition is the deposition of GO nanosheets and crosslinkers in a layer-by-layer manner. The crosslinkers can react and/or interact with GO nanosheets to construct the GO framework. The membrane substrate is immersed in the GO dispersion and crosslinker solution one by one in several cycles to form several bilayers. Some typical crosslinkers are organic molecules and positively charged electrolytes.^12–14^

Due to their good chemical, mechanical and thermal properties as well as precise sieving capability, GO-based membranes have been applied in gas, water and organic solvent separations. For gas separations, as the membrane is in the dry state, the interlayer spacing or *d*-spacing of GO nanosheets is ∼0.3 nm, which is suitable to separate small gas molecules from large ones.^10,20^ Since GO contains hydroxyl and carboxyl functional groups, adsorption of CO_2_ is enhanced which may retard or promote the CO_2_ transport rate depending on the microstructure of the GOM.^7^ In water and organic solvents, the interlayer spacing of GO can be enlarged or swelled by the water and organic solvent molecules to at least 0.7 nm, depending on the polarity and affinity of the solvent.^21,22^ This makes GOMs suitable for separating divalent salts by nanofiltration,^23,24^ organic solutes by organic solvent nanofiltration^25–27^ and water–organic solvent mixtures by pervaporation.^28,29^ Various methods have been developed to restrict the swelling of the GO framework, such as crosslinking, intercalation and physical confinement. In addition to restricting the swelling, these methods can microscopically tune the structure of the GO framework, resulting in notable changes in membrane properties and separation performance.

This review aims to summarize state-of-the-art publications within the last 3 years on microscopically tuning GO nanosheets and its effect on the macroscopic properties and separation performance of GOMs. We first briefly discuss the transport mechanism and recent findings on solvent transport in GOMs. Next, publications on tuning the structure and modifying the chemical composition of the GO framework will be elaborated on. As there are many publications focused on modulating the interlayer spacing of the GO framework, this part is further elaborated based on different crosslinkers and intercalators. Besides, controlling the size and modifying the functional groups of GO nanosheets will also be discussed. Finally, a summary of recently developed GO-based membranes and the outlook is given.

## 2. Transport mechanism

**Fig. 2:**
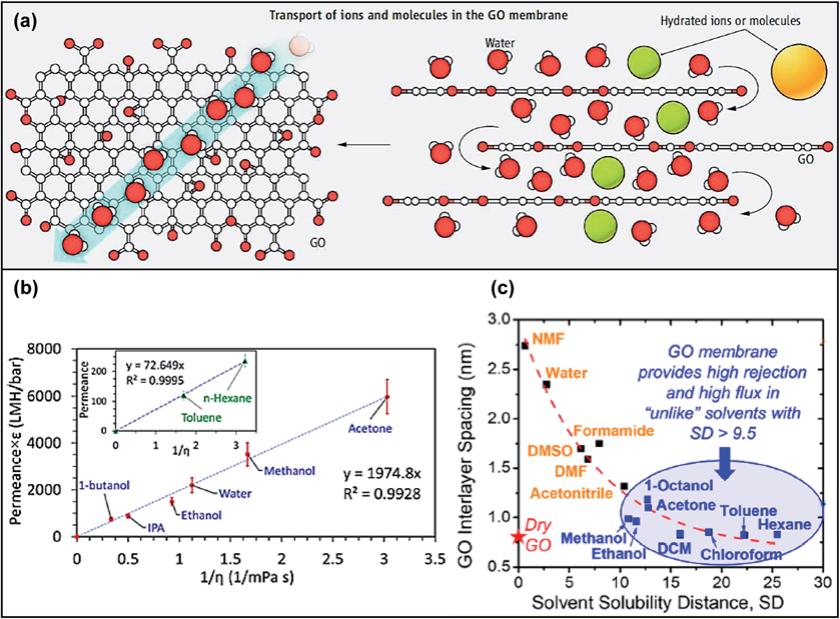
(a) Illustration of the transport mechanism in the GOM. Adapted from ref. 30 with permission from the American Association for the Advancement of Science, copyright 2014. (b) Correlation of solvent permeances with dielectric constant *ε* and solvent viscosity *η*. Adapted from ref. 21 with permission from the American Chemical Society, copyright 2018. (c) Correlation of the GO interlayer spacing with the solvent solubility distance of GO and solvent. Adapted from ref. 22 with permission from the American Chemical Society, copyright 2020.

The transport mechanism of GOMs is dominated by size exclusion from the laminar structure. The in-plane pore size and charge effect also influence the separation performance of GOMs to a certain degree. Early studies on GOMs illustrated that the transport of molecules in stacked GO nanosheets followed the interconnected tortuous nanochannels between the GO nanosheets,^30^ as shown in Fig. 2a. Solutes with diameters smaller than the interlayer spacing of the GO framework could permeate through the GOM and large solutes would be blocked. According to a simulation study conducted with a double-layer GOM, the bulk behavior (no separation) occurred when the *d*-spacing increased above 0.9 nm for H_2_ and 1.0 nm for CH_4_ and N_2_.^31^

The *d*-spacing of GO is varied in the dry state and in different solvents. The *d*-spacing between GO nanosheets in a dried state is ∼0.3 nm, which can only allow the passage of small gas and water molecules.^30^ The GO framework will be swollen in different solvents according to their affinity and dielectric constants, resulting in an increased *d*-spacing. Majumder *et al.* investigated the solvent permeance across a GOM and found that the interlayer spacing of GO layers could be correlated with the dielectric constant of the organic solvents with a power-law (0.48) dependence, which originated from electrostatic interactions between GO layers in the solvents.^21^ The solvent permeance was also linearly fitted to the reciprocal product of solvent viscosity and dielectric constant, as depicted in Fig. 2b. In a later study, Mi's group found that the Hansen solubility difference between GO and solvents could estimate the GO swelling and interlayer spacing. Solvent with a small solubility distance, which indicated a good affinity towards GO, could increase the interlayer spacing significantly and *vice versa* (Fig. 2c).^22^ Although the conclusions of these two studies were not the same, both showed that the interlayer spacing could significantly affect the transport behavior of GOMs. Therefore, microscopically tuning the structure of the GO framework plays an important role in the membrane performance. In the following sections, various methods to microscopically tune the GOM performance will be discussed in detail.

## 3. Tuning the structure of the GO framework

As mentioned above, the architecture and the perpendicular distance between adjacent GO lattices, *i.e.*, *d*-spacing, are key factors to determine the molecule separation performance of the membranes.^32^ Fine tuning of *d*-spacing as well as the nanochannel size enables the GOMs to sieve molecules and to block solutes with kinetic diameters larger than nanochannels.^33,34^ The use of chemical and physical modification methods to modulate *d*-spacing and to enhance the stability and functionality of GOMs has increased in recent years. In this section, several modification methods, including covalent crosslinking, ionic interaction, and compositing with polymers, nanoparticles or 2D materials, for tuning the structure of GO frameworks and the membrane properties, will be introduced. Table 1 summarizes the separation performance of GOMs with different intercalates.

**Table 1:** A comparison of GOMs with different intercalates

Intercalate type	Intercalate	Application	Permeability	Rejection or selectivity	Ref.
Covalent cross-linking	Diamines	Pervaporation	2297 g m^−2^ h^−1^	Water concentration: 99.8 wt%	28
	Diamines	Pervaporation	19.7 kg m^−2^ h^−1^	Ion rejection: 99.9% (3.5 wt% seawater at 90 °C)	37
	Amine-terminated polyamidoamine dendrimers	Water desalination	Water: 124 kg m^−2^ h^−1^; butanol: 9108 g m^−2^ h^−1^	NaCl rejection: >99.99%	40
	Interfacial molecular bridges	Water purification	Water: 7.6–8.1 L m^−2^ h^−1^ bar^−1^	Dye rejection: 98.3%–98.9%	41
	Cysteamine	Gas separation	H_2_: 51.5 × 10^−6^ cm^3^ (STP)/(cm^2^ s cmHg)	H_2_/CO_2_: 21.3	42
	Polyethyleneimine	Nanofiltration	Water: 67.5–72.2 L m^−2^ h^−1^ bar^−1^	Crystal violet and Victoria blue B rejection: >99%	43
Polymer composite	PEG	Oil/water separation	Water: 4890 L m^−2^ h^−1^	Oil: 100%	45
	PDMAEMA	Nanofiltration	Water: 62.61 L m^−2^ h^−1^ MPa^−1^	Congo red and methylene blue rejection: >95%	46
	Polyvinylidene fluoride-*g*-poly(*N*-isopropylacrylamide)	Water purification	Water with bovine serum albumin: 113.4 L m^−2^ h^−1^ bar^−1^	Bovine serum albumin rejection: 82.5%	47
	Lignin	Nanofiltration	Water: 1182 L m^−2^ h^−1^ bar^−1^	Rhodamine B rejection: ∼100%	49
	PAN-GPs	Water purification	Cu-EDTA feed solution: 14.6 L m^−2^ h^−1^	Cu-EDTA rejection: 99.2%	50
	Polypyrrole	Nanofiltration	Water: 21.14 L m^−2^ h^−1^ bar^−1^	Crystal violet, eriochrome black T, Congo red, and trypan blue rejection: >99%	51
Nanoparticle	SiO_2_ (*in situ*)	Organic solvent nanofiltration	Methanol: 290 L m^−2^ h^−1^ bar^−1^	Rose bengal: 91.9%; methylene blue: 45.8%	55
	SiO_2_	Nanofiltration	Water: 44.2 L m^−2^ h^−1^ bar^−1^	Eosin Y: 97.2%; methyl orange: 91.0%	56
	EDA-SiO_2_	Oil/water separation	Water: 330 L m^−2^ h^−1^ bar^−1^	Oil: 99.4%	57
	Fe_3_O_4_ (*in situ*)	Nanofiltration	Water: 296 L m^−2^ h^−1^ bar^−1^	Rhodamine B: 98%	52
	NH_2_-Fe_3_O_4_	Nanofiltration	Water: 15.6 L m^−2^ h^−1^ bar^−1^	Congo red: 98%; methylene blue: 70%; NaCl: 15%	58
	POSS-NH_2_	Gas separation	CO_2_: 16.5 × 10^−6^ cm^3^ (STP)/(cm^2^ s cmHg)	CO_2_/CH_4_: 74.5	59
	ZIF-8 (*in situ*)	Organic solvent nanofiltration	Methanol: ∼6800 L m^−2^ h^−1^ bar^−1^	Rose bengal: 97%; rhodamine B: 57%; methylene blue: 15%	60
	ZIF-8 (ice templating and *in situ*)	Nanofiltration	Water: 60 L m^−2^ h^−1^ bar^−1^	Methyl blue: 99%; vitamin B12: 90%	61
Cation modification	Li^+^, Na^+^, K^+^, Ca^2+^ or Mg^2+^ ions	Ion sieving and separation	Water: 0.1–0.36 L m^−2^ h^−1^	Ion rejection: >99%	62
	K^+^ ion	Molecular separation and water purification	Water: 0.47 L m^−2^ h^−1^	Mg^2+^ selectivity: 97.5%	63
2D material	Triazine-based COF	Nanofiltration	Water: 226.3 L m^−2^ h^−1^ bar^−1^	NaCl: 95.5%; methyl blue: 93.3%	65
	COF, TpPa	Gas separation	H_2_: 1.067 × 10^−6^ mol m^−2^ s^−1^ Pa^−1^	H_2_/CO_2_: 25.57	66
	Glycine and g-C_3_N_4_	Nanofiltration	Water: 207 L m^−2^ h^−1^ bar^−1^	Methylene blue: 87%; Evans blue: 99%	67
	g-C_3_N_4_	Nanofiltration	Water: 15.4 L m^−2^ h^−1^ bar^−1^	Methylene blue: 92.6%; methyl orange: 41.2%	68
	TiO_2_ nanosheets	Nanofiltration	Water: 9.36 L m^−2^ h^−1^ bar^−1^	Methylene blue: 98.8%; methyl orange: 97.3%	69
Horizontal membrane	Heat treatment	Gas separation	H_2_: 2253 × 10^−6^ cm^3^ (STP)/(cm^2^ s cmHg)	H_2_/CO_2_: 6.7	70
	Physical confinement	Water desalination	Water: 2–3 L m^−2^ h^−1^ bar^−1^	NaCl: 97%	71

### 3.1 Covalent crosslinking

GO is used as a building block for the preparation of laminar membranes because GO contains functional groups such as carboxyl, hydroxyl, and epoxy groups, which allows GO to exhibit high hydrophilicity and the capability to be chemically modified. Covalent crosslinking using small molecules or polymers is one of the most explored methods to tune the nanochannel sizes and to improve the molecular transport properties of GOMs.^35,36^ Crosslinked GOMs also exhibit superior durability and mechanical properties. The use of crosslinkers such as diamine, dopamine and polyethyleneimine, with varied chain lengths and functionalities, can modulate the *d*-spacing, ionic strength and elastic moduli, leading to selective and effective molecular separations.

**Fig. 3:**
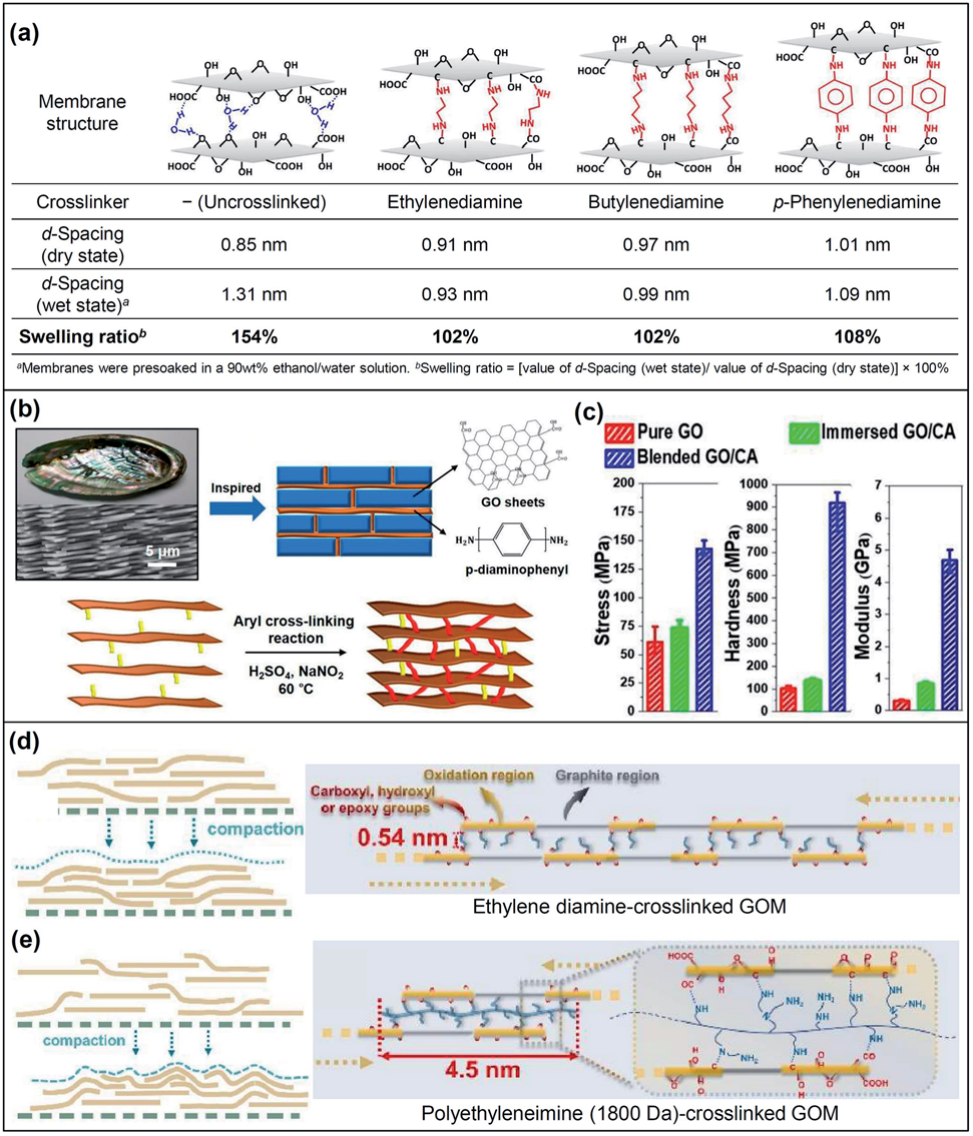
(a) Structural diagram and properties of diamine-crosslinked GO membranes. Adapted with permission from ref. 28. American Chemical Society, Copyright 2014. (b) Schematic illustration and synthetic process of the nacre-mimetic membrane with GO and *p*-phenylenediamine. (c) Stress, hardness and elastic modulus of the nacre-mimetic crosslinked GO membrane. CA: crosslinker. Adapted with permission from ref. 38. American Chemical Society, copyright 2019. Schematic illustrations of the GO stacking structure in the (d) ethylene diamine-crosslinked GO and (e) polyethyleneimine (1800 Da)-crosslinked GO membranes. Reproduced with permission from ref. 43. Elsevier Science Ltd., copyright 2021.

Diamines are commonly used as the crosslinkers for GO lattices *via* nucleophilic additions between amines and the epoxide or carboxyl groups of GO. Hung and co-workers used diamines with different spacer arm lengths and structures to fabricate crosslinked GOMs.^28^ The results demonstrated that the *d*-spacing value and the swelling ratio of GOMs could be modulated *via* diamine crosslinking (Fig. 3a). The increment of lengths and bulkiness of the crosslinker spacer arms led to the enlargement of the *d*-spacing. The crosslinked GOMs demonstrated relatively small swelling ratios, indicating that the crosslinked membranes had improved resistance to lattice stretching. The membrane also showed high operation stability during long-term operation at 30 °C for 120 h. Zhou *et al.* prepared a series of crosslinked GOMs using several aliphatic terminal diamines with different spacer arm lengths.^37^ The *d*-spacing of the uncrosslinked and crosslinked GOMs could be finely tuned from 0.85 nm to 1.23 nm. The permeability and stability of membranes with different *d*-spacing values were systematically investigated. The results indicated that the 1,4-diaminobutane-crosslinked GOM with 1.05 nm *d*-spacing exhibited superior performance with a water flux of 19.7 kg m^−2^ h^−1^ and 99.9% ion rejection at 90 °C for desalination of 3.5 wt% seawater. The crosslinked GOM could be operated for seawater desalination for up to 168 h at 75 °C, suggesting its high stability. Recently, bio-inspired nacre-like GOMs have been gaining attention due to their outstanding mechanical properties. Han's group synthesized a covalently conjugated GO with *p*-phenylenediamine as the crosslinker based on the “brick-and-mortar” concept of nacre (Fig. 3b).^38^ After crosslinking, the *d*-spacing of the GOM decreased to 0.61 nm from 0.64 nm for the uncrosslinked GOM, suggesting that *p*-phenylenediamine could easily cooperate with the GO lattices and thus reduce the *d*-spacing. Compared to the mechanical properties of the pristine (uncrosslinked) GOM, this nacre-like crosslinked GOM exhibited a 2.3-fold increase in tensile strength (142.9 ± 6.4 MPa), 15.7-fold increase in modulus, and 9.0-fold increase in hardness (Fig. 3c). Aubin-Tam's group also found that γ-poly(glutamic acid) and calcium ions were applicable as crosslinkers to fabricate nacre-like crosslinked GOMs.^39^ Similarly, the crosslinked GOMs possessed a high strength of 150 ± 51.9 MPa and modulus of 21.4 ± 8.7 GPa, showing a 2.2-fold and over 1.7-fold increase, respectively, with respect to pristine GOMs.

Apart from using diamines as the crosslinker, dendrimers and dopamine are also reported to be effective crosslinkers for tuning the *d*-spacing of GOMs. Dendrimers are highly branched macromolecules with radially symmetric structures and nanometer-scale sizes. Jiang's group employed primary amine-terminated polyamidoamine dendrimers as the crosslinkers to tune the *d*-spacing of GOMs.^40^ Dendrimers with radii of gyration of 0.68 nm, 1.04 nm and 1.16 nm were synthesized and incorporated with GO nanosheets to generate crosslinked GOMs with well-defined interlaminar structures and high mechanical stability. The *d*-spacing of the crosslinked GOMs was able to be tuned in the range of 0.43 nm to 0.76 nm in the wet state. The resulting GOMs exhibited a permeation flux of 124 kg m^−2^ h^−1^ and excellent separation performances for seawater desalination and butanol dehydration. Liu, Jin and their co-workers reported a molecular bridge strategy to fabricate robust GOMs.^41^ Polydopamine served as a short-chain crosslinker to bridge GO lattices, which enabled the GO lattices to show high resistance against swelling. Meanwhile, aldehyde-modified chitosan was used as an interfacial long-chain molecular bridge to connect the GO layer and the porous substrate. The obtained GOM displayed superior stability under cross-flow, high-pressure (up to 10 bar), and long-time operation conditions for water-based separations. Jin's group also employed cysteamine, which contains an amino group and a thiol group to react with the oxygen-containing groups on GO, to prepare cysteamine-crosslinked GOMs.^42^ The *d*-spacing was tuneable between 0.91 nm and 0.98 nm *via* using different ratios of cysteamine and GO. The crosslinked GOM with a *d*-spacing of 0.94 nm exhibited significantly improved size discrimination properties and doubled the H_2_/CO_2_ selectivity in comparison with that of the uncrosslinked GOM. Recently, Shao and co-workers investigated the separation performance of crosslinked GOMs using ethylene diamine and polyethylenimines with different molecular weights of 600 Da, 1800 Da and 10 000 Da.^43^ The results showed that the small molecular crosslinker, *i.e.*, ethylene diamine, facilitated the in-plane GO stacking while the polyethyleneimine crosslinker was found to inhibit the aligned stacking of adjacent GO lattices (Fig. 3d and e). The results showed that the GOM using the polyethyleneimine crosslinker with 1800 Da molecular weight demonstrated a superior water permeance ranging from 67.5 to 72.2 L m^−2^ h^−1^ bar^−1^, which was 5 times higher than that of the pristine GOM.

### 3.2 Polymer–GO composites

**Fig. 4:**
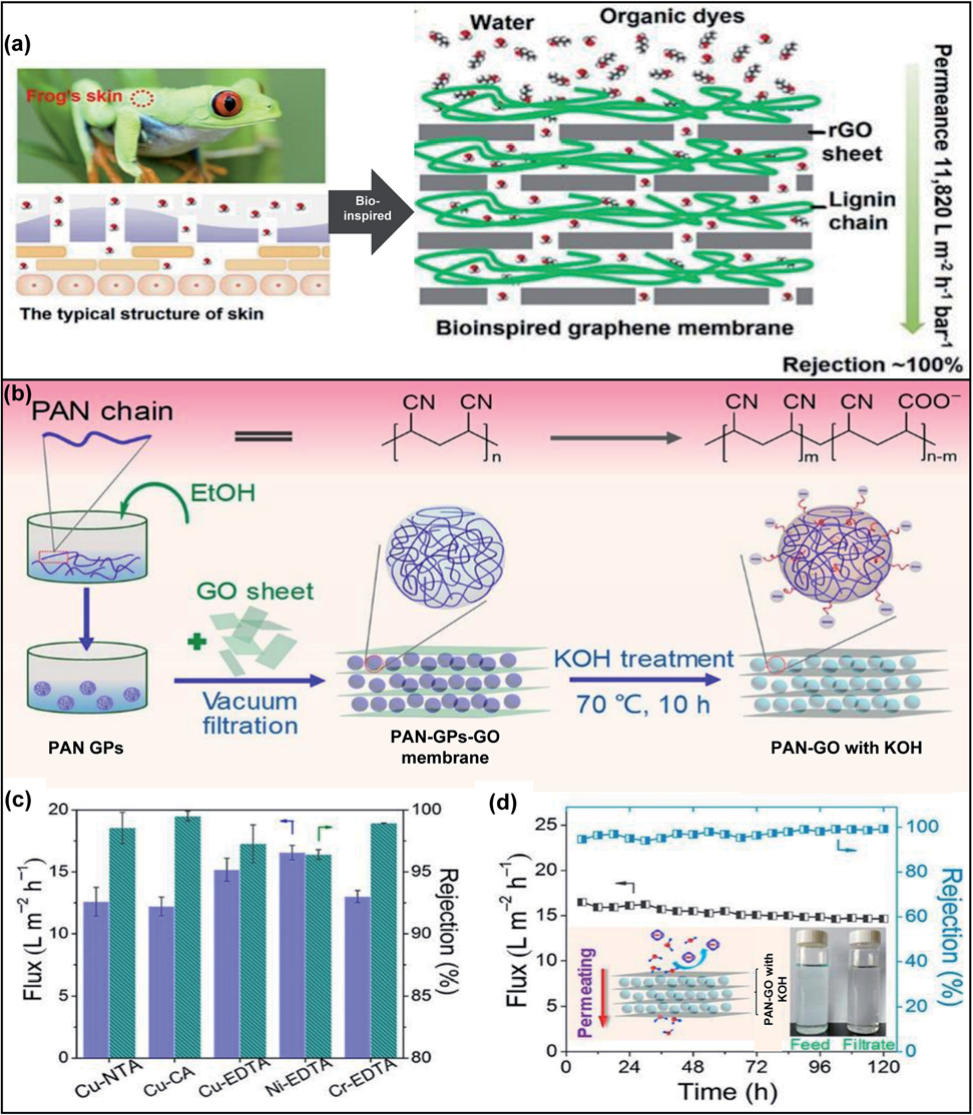
(a) Schematic diagram for the mechanism of nacre-like GO–lignin composited membranes. The membrane structure is inspired by a frog's skin. (b) Schematic illustration of intercalating PAN-GPs to fabricate the PAN-GP-GO membrane. (c) The fluxes and rejections for heavy metal–organic complex anions. NTA: nitrilotriacetic acid; CA: citric acid; EDTA: ethylenediaminetetraacetic acid. (d) Variation of the flux and rejection for Cu-EDTA by the PAN-GO membrane with KOH treatment. Insets: schematic illustration of Cu-EDTA solution permeating behavior and image of the above solutions before and after filtration. Adapted with permission from ref. 49 and 50. American Chemical Society, copyright 2020.

By engineering GO with polymers to form nanocomposite membranes, the *d*-spacing and functionality of GOMs can be modulated. Two main strategies have been developed to incorporate GO within a polymer matrix. The first method is to disperse GO nanosheets in a monomer solution, followed by *in situ* polymerization. The other method is to blend GO nanosheets with a polymer solution for membrane casting. Various polymers, such as polyethylene glycol (PEG), polymethacrylates, and polyacrylamides, have been used to integrate GO nanosheets into a polymer matrix.^44^ Jiang *et al.* fabricated reduced GO (rGO) aerogel membranes *via* reduction-induced self-assembly and hydrogen bonding interactions.^45^ The authors incorporated oxygen-containing PEGs with GO nanosheets. The ether groups of PEG as hydrogen bond acceptors could form relatively strong hydrogen bonds with the phenolic hydroxyl and carboxyl groups of GO nanosheets. Therefore, the introduction of PEG led to the decrement of the GO laminate sizes and the structural shrinkage of the GO network, resulting in membranes with smaller pore sizes and high porosity up to 96%. The *d*-spacing of GOMs could be tuned from 0.33 nm to 0.62 nm by using PEGs with different molecular weights. The obtained GOM could reject oil-in-water emulsions with different sizes and exhibited water flux up to 4890 L m^−2^ h^−1^ under 0.1 bar. Zhao *et al.* reported nanofiltration membranes with gas-controlled charge-gated channels for switchable rejection towards both cations and anions.^46^ The membranes were prepared by assembling poly(*N*,*N*-dimethylaminoethyl methacrylate) (PDMAEMA), which is a CO_2_-responsive polymer, on the surface of GO nanosheets. The obtained GO–PDMAEMA membrane exhibited reversibly switchable surfaces carrying positive and negative charges upon addition and removal of CO_2_. Although the *d*-spacings of GO sheets were the same (0.86 nm) when the charges got switched, the membranes showed high rejection towards MgCl_2_ when it carried positive charges and high rejection towards Na_2_SO_4_ when it was negatively charged due to the Donnan effect. Another study of thermal-responsive GO–polymer composite membranes was performed using polyvinylidene fluoride-*graft*-poly(*N*-isopropylacrylamide) as the matrix.^47^ The thermally responsive nature of poly(*N*-isopropylacrylamide) allowed the tunability of pore size and water permeance at different temperatures. Recently, bio-inspired strategies are applied to design and fabricate GOMs with ordered nanostructures and enhanced permeability and stability.^48^ Ding *et al.* reported a bioinspired membrane using hydrophilic polymer lignin and rGO nanosheets for water transport and separation of organic dyes (Fig. 4a).^49^ The *d*-spacing of the rGO framework was around 0.71 nm and it was increased to 1.04 nm after incorporation with lignin. The lignin–GO composite membrane demonstrated a significantly high water permeance of 1182 L m^−2^ h^−1^ bar^−1^ as well as excellent separation efficiency for the separation of organic dyes.

Recently, soft polymeric nanoparticles have been attracting increasing attention to intercalate GOMs for the improvement of separation performance. The soft nanoparticles exhibit superior (i) surface functionality compared to those of small molecular crosslinkers or metal ions and (ii) deformability in contrast to hard inorganic particles, which facilitates the formation and stability of nanochannels. Zhang's group reported that the *d*-spacing of the GOM was tuneable *via* the intercalation of soft polyacrylonitrile gel particles (PAN-GPs) between the GO nanosheets.^50^ The preparation procedure is displayed in Fig. 4b. PAN-GPs had flexible shapes and underwent deformation upon application of external pressure. Additionally, the alkaline treatment could also enhance the negative charge and hydrophilicity on the PAN-GP surfaces. The *d*-spacing of the GOM containing PAN-GPs was able to be tuned by controlling the swelling of the deformed PAN-GPs, anion–π interaction, and external pressure. The *d*-spacing of the pristine GOM, the GOM with PAN-GPs and the GOM with PAN-GPs and KOH solution treatment increased from 0.83 nm to 0.86 nm and further to 0.92 nm, respectively. The GOM with PAN-GPs exhibited fast and selective water permeation to separate heavy metal–organic complexes containing copper (Cu), nickel (Ni) or chromium (Cr) ions with over 96% rejection (Fig. 4c). The membrane could effectively separate copper ethylenediaminetetraacetate (Cu-EDTA) from water with a permeating flux 4–13 times higher than those of other reported 2D membranes. The permeating flux slightly declined by 8% from 15.9 to 14.6 L m^−2^ h^−1^ after operating for 120 h, indicating its high stability and durability (Fig. 4d). Similarly, Ou and co-workers intercalated soft polypyrrole nanoparticles into GOMs for tuning their *d*-spacing and enhancing their properties.^51^ The relatively strong interaction between GO and polypyrrole nanoparticles improved the membrane's mechanical stability and further reduced the *d*-spacing from 1.41 nm to 1.27 nm, leading to the enhancement of separation ability towards nano-sized dyes. The strong dye adsorption ability of polypyrrole also increased the dye molecule rejection from 60% for the pristine GOM to 97% for the GO–polypyrrole membrane after the initial filtration treatment.

### 3.3 Nanoparticle–GO composites

Nanoparticles, with a large diversity, have been employed as good spacers for the GO framework due to their rigid structure. Previously, nanoparticles were incorporated with GO nanosheets *via* physical mixing, resulting in non-uniform dispersity and an unstable structure.^52–54^ Recent studies focus on (i) modifying the nanoparticles to have high affinity with GO and (ii) controlling the position of the nanoparticles in the GO framework *via in situ* synthesis.

**Fig. 5:**
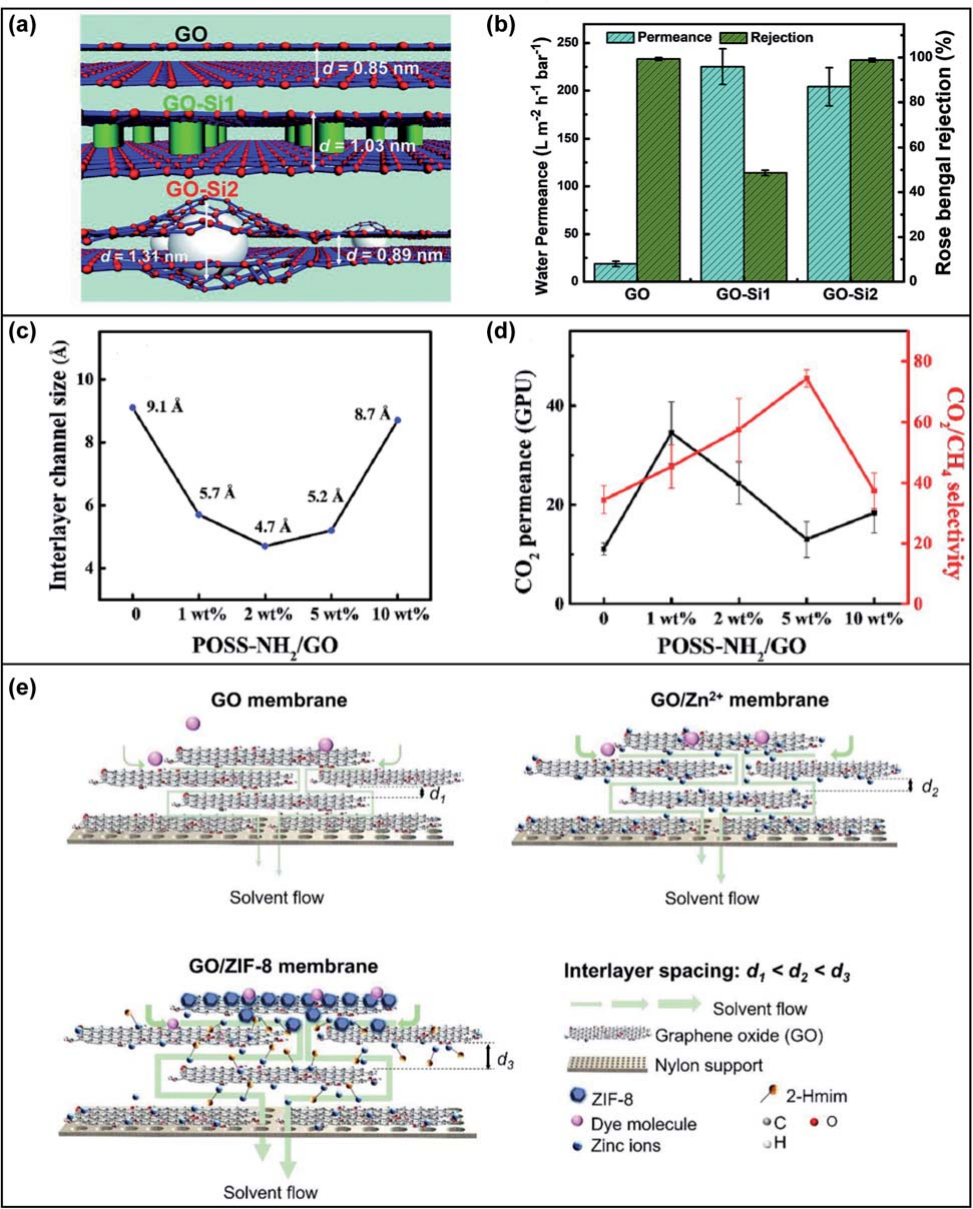
(a) Schematic illustration of the *d*-spacing for GO, GO-Si1 and GO-Si2 membranes. GO was the pristine GO membrane, GO-Si1 was fabricated from GO nanosheets with the attachment of APTES and GO-Si2 was fabricated by filtering NaOH solution through GO-Si1. (b) The water permeance and rose bengal rejection of the GO, GO-Si1 and GO-Si2 membranes. Adapted with permission from ref. 55. The Royal Society of Chemistry, copyright 2019. (c) The relationship between the interlayer channel size and the loading of POSS-NH_2_. (d) The CO_2_ permeance and CO_2_/CH_4_ selectivity of the developed membrane with different POSS-NH_2_ loadings. Adapted with permission from ref. 59. Elsevier Science Ltd., copyright 2020. (e) Schematic illustration of the interlayer spacing and solvent flow of GO, GO/Zn^2+^ and GO/ZIF-8 membranes. Adapted with permission from ref. 60. Elsevier Science Ltd., copyright 2021.

Nunes's group applied an *in situ* method to synthesize silicon oxide (SiO_2_) nanoparticles during the formation of a GOM.^55^ (3-Aminopropyl)triethoxysilane (APTES) as the precursor of silica was added to the GO solution before vacuum filtration. APTES could uniformly attach to the GO nanosheets by forming hydrogen bonds with GO. After treatment with NaOH aqueous solution, the SiO_2_ nanoparticles were generated between the GO layers. The *in situ* synthesized SiO_2_ particles were covalently bonded to the GO layers and slightly increased the *d*-spacing of GO nanosheets (Fig. 5a). The fabricated membrane had a 10-fold increase in water permeance without sacrificing rose bengal rejection thanks to the dual-spacing channels generated by SiO_2_ nanoparticles (Fig. 5b). Similar results have also been reported by directly incorporating SiO_2_ nanoparticles into GOMs. A tent-shaped structure was formed on the membrane surface and the *d*-spacing increased slightly with the increasing content of SiO_2_ (mass ratio of SiO_2_ : GO < 1).^56^ When the mass ratio of SiO_2_ : GO > 1, the X-ray diffraction (XRD) peak was broadened due to the amorphous SiO_2_. The water flux increased with SiO_2_ loading but the rejections towards small solutes, such as methyl orange and *p*-hydroxybenzoic acid, dropped significantly. In order to restrict the enlargement of *d*-spacing between GO nanosheets, a group of researchers added ethylenediamine to crosslink GO nanosheets with SiO_2_.^57^ Unlike the aforementioned studies, the *d*-spacing did not show an obvious change upon increasing the SiO_2_ content due to the crosslinking of ethylenediamine. The water flux for separating oil/water was enhanced due to the hierarchical porous nanostructure and SiO_2_ induced large pores.

Besides SiO_2_, other nanoparticles were also intercalated into the GO framework, but some of them may not be able to interact with GO nanosheets, resulting in non-uniform dispersion. It is promising to apply an *in situ* method and/or functionalize nanoparticles to improve the affinity and dispersibility. An *in situ* method to synthesize and intercalate nanoparticles (Fe_3_O_4_, UiO-66 and TiO_2_) into GO nanosheets was developed.^52^ The *in situ* fabrication method resulted in a uniform distribution of nanoparticles and a stable structure for water filtration. The water permeance and dye rejection of the *in situ* synthesized Fe_3_O_4_-decorated rGO membrane were much higher than those of the Fe_3_O_4_/rGO membrane fabricated *via* the physical mixing method. In another study, amino-functionalized iron oxide (Fe_3_O_4_) nanoparticles were synthesized and inserted into the GO framework.^58^ The *d*-spacing increased with the loading of NH_2_-Fe_3_O_4_ from 7.9 Å to 8.8 Å. As a result, the water flux increased dramatically with the increase of NH_2_–Fe_3_O_4_ loading, but the rejection towards NaCl and Na_2_SO_4_ dropped largely due to the enlarged nano-channel and loosened structure. In addition to solid nanoparticles, porous nanoparticles, such as polyhedral oligomeric silsesquioxane (POSS) and metal–organic frameworks (MOFs), were also be employed as intercalators for GOMs. The amino functionalized POSS was able to crosslink the GO nanosheets and restrict the swelling in the humidified state.^59^ The interlayer channel size was found to decrease first and then increase with the loading of POSS-NH_2_ (Fig. 5c). However, the CO_2_ permeance increased as the facilitated transport induced by the NH_2_ group on POSS offset the decrease in channel size (Fig. 5d). Both the sterically hindered interlayer channel and NH_2_ facilitated transport led to the enhanced separation of CO_2_/CH_4_.

Due to the porous structure and tunable pore size, MOFs have been investigated as effective intercalators for the GO framework. Recently, two studies reported graphene oxide membranes intercalated with zeolitic imidazolate framework-8 (ZIF-8) *via* an *in situ* method. Huang *et al.* added zinc nitrate (Zn(NO_3_)_2_) into a GO solution and fabricated a GOM *via* vacuum filtration.^60^ The ligand solution was then filtered through the membrane to generate ZIF-8 particles. The interlayer spacing of the GOM was found to be enlarged, leading to a significant increase of the methanol permeance to 6800 L m^−2^ h^−1^ bar^−1^ (Fig. 5e). The developed GOM demonstrated high rejections towards rose bengal (∼97%) and reactive black 5 (∼98%), but low rejections (∼20%) towards methylene blue and methyl orange. Elimelech, An and co-workers prepared a ZIF-8-hybridized GOM by an ice templating and *in situ* crystallization method.^61^ They first fabricated the GOM on ceramic tubes and freeze-dried it *via* the ice templating technique. The freeze-dried GOM was then immersed into a ZIF-8 precursor solution and subsequently treated with MeOH/NH_3_ H_2_O to control the growth of ZIF-8 along the edges of the GO nanosheets. The *d*-spacing increased from 0.75 nm to 0.93 nm by freeze-drying and remained fixed after the intercalation of ZIF-8. The growth of ZIF-8 at the edges of GO nanosheets was validated using low-field nuclear magnetic resonance, where a new population of small pores appeared possibly representing the vacancies at the edges of GO nanosheets. Compared to the pristine GOM, the water flux of the ZIF-8 intercalated GOM was enhanced by more than 10 times with a slightly higher rejection towards methyl blue (molecular weight ∼ 800 Da) attributed to the steric hindrance of ZIF-8.

### 3.4 Cation-modified GOMs

**Fig. 6:**
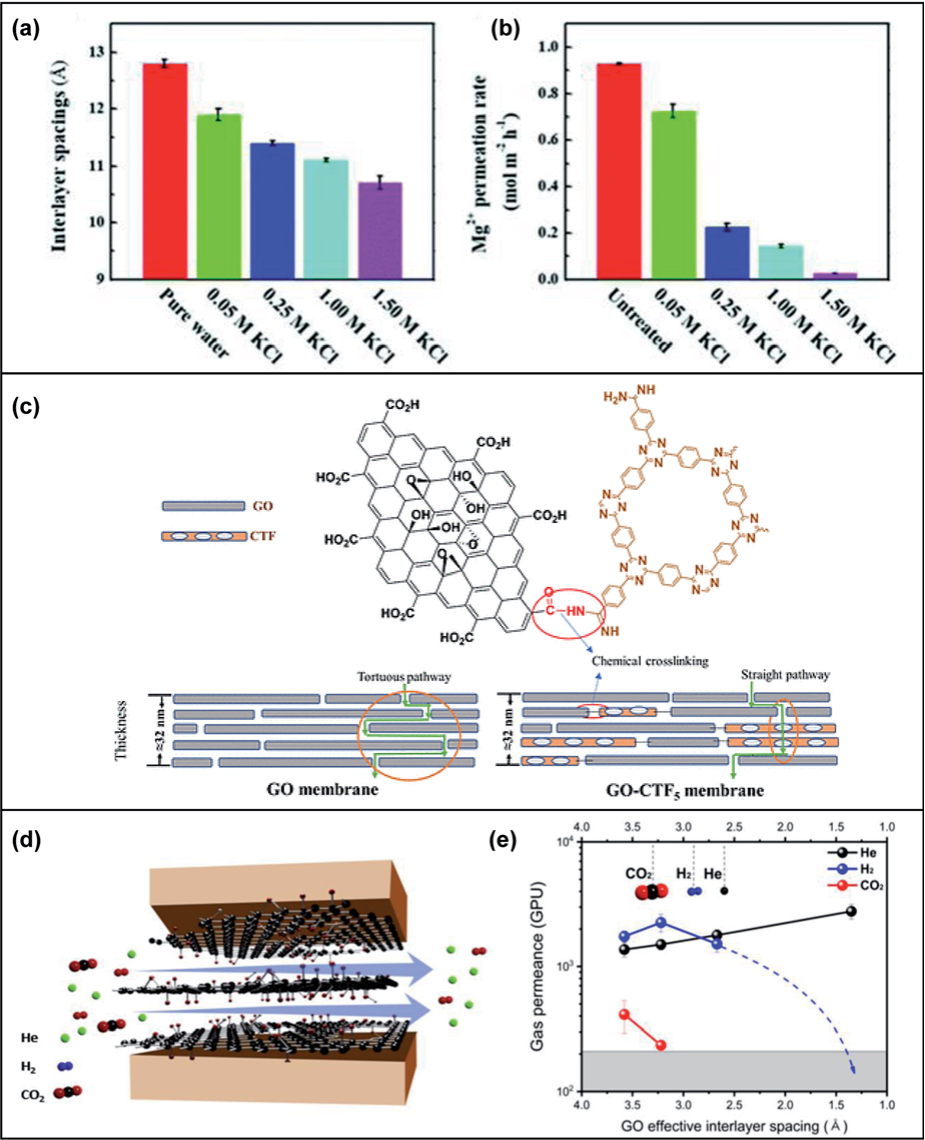
(a) A bar graph depicting the interlayer spacings in angstroms when the GO membranes are immersed in pure water as compared to in KCl solutions (0.05 M to 1.50 M). (b) A bar graph depicting the Mg^2+^ permeation rates of untreated GO membranes as compared to treated membranes in KCl solutions (0.05 M to 1.50 M). Adapted with permission from ref. 63. Royal Society of Chemistry, copyright 2020. (c) A schematic illustration of the chemical crosslinking between GO and CTF and the transport pathway in the GO membrane and GO-CTF mixed membrane. Reproduced from ref. 65. American Chemical Society, copyright 2019. (d) A scheme illustrating the direction of gas permeation horizontally along the planes of graphene sheets. (e) The relationship of the gas permeation rate with interlayer spacing. Adapted from ref. 70. Elsevier Science Ltd, copyright 2020.

Cation–π interactions are noncovalent molecular interactions between a positively charged molecule and an electron-rich π system. Cation–π interactions play an important role in constructing biological structures, molecular recognition and catalysis. The strength of cation–π interactions is influenced by the nature of the cation and π systems, binding geometry and environmental polarity. Exploiting cation–π interactions, cations have been used to react with GO nanosheets for manipulating the *d*-spacings of GOMs to the angstrom level. In 2017, Chen *et al.* pioneered the use of cations to precisely control the *d*-spacing in GOMs using various cations (Li^+^, Na^+^, K^+^, Ca^2+^ or Mg^2+^ ions). The *d*-spacing can be tuned down to 1 Å.^62^ Recently, Liang *et al.* found that the *d*-spacings could be tuned to a range of 11.9 Å to 10.7 Å in potassium chloride (KCl) solutions (Fig. 6a).^63^ Despite the thickness of the investigated membranes, the lowest permeation rate and highest rejection for Mg^2+^ were achieved, as demonstrated in Fig. 6b. By adjusting Na^+^, Li^+^ and K^+^ concentrations, ion rejection of these membranes could be controlled due to the strong cation–π interaction with the GOMs. Bae and co-workers also investigated the cationic effect on tuning the *d*-spacing of GOMs and applied the membranes for H_2_/CO_2_ separation.^64^ The developed GOMs, which were intercalated and crosslinked with lanthanum (La^3+^) or cobalt (Co^2+^) cations between the GO layers, could effectively separate H_2_ gas from a H_2_/CO_2_ mixture. However, unlike the study by Liang's group, the H_2_/CO_2_ selectivity of the cation-treated membranes varied based on their thickness.

### 3.5 2D material–GO composites

As GO is a flexible 2D material, it can be stacked with other 2D materials, such as covalent organic frameworks (COFs), graphitic carbon nitride (g-C_3_N_4_) and 2D titanium dioxide (TiO_2_) sheets, to form a hybridized stacked membrane.

COFs are a group of 2D materials possessing intrinsic uniform pores with large porosities. An NH_2_-functionalized covalent triazine framework (CTF-1) was grafted onto GO nanosheets with different ratios and then stacked into mixed sheet membranes for evaluation.^65^ Fourier-transform infrared spectroscopy (FTIR) and X-ray photoelectron spectroscopy (XPS) results indicated that the CTF was chemically linked to GO nanosheets *via* the carboxyl groups of GO forming a large hybrid sheet. The incorporation of CTF created large interplanar pores and thus decreased the transport path, as illustrated in Fig. 6c. The optimized membrane exhibited a 12-fold higher water flux than pure GOMs with slightly lower rejection towards organic dyes. Another study directly mixed a type of COF, TpPa, with a GO aqueous solution to fabricate a membrane.^66^ Hydrogen bonds might be formed between the GO and COF to form a robust and stable membrane. The H_2_/CO_2_ selectivity of the COF/GO composite membrane was enhanced with a higher permeance of H_2_ compared to the pristine GOM.

g-C_3_N_4_ is a 2D material formed by carbon and nitrogen sp2 hybridization. Like COFs, it possesses inherent nanopores with ∼0.3 nm size, which can allow water to pass through. Zhan *et al.* combined g-C_3_N_4_ and GO nanosheets with the assistance of glycine to enhance their interaction.^67^ Both g-C_3_N_4_ and glycine increased the interlayer spacing, but g-C_3_N_4_ narrowed the nanochannels while glycine increased the dimensions of the nanochannels. The water permeance decreased slightly when only g-C_3_N_4_ was intercalated. In contrast, the water permeance was increased to 4-fold if glycine was incorporated into the GO/g-C_3_N_4_ composite membrane. Wang's group utilized g-C_3_N_4_ to intercalate GO nanosheets with different loadings.^68^ From XRD results, the authors proposed that the g-C_3_N_4_ nanosheets might incline an unorderly manner between the GO nanosheets instead of layer by layer. The composite membrane exhibited a 2-fold higher water permeance than the pure GOM with a comparable rejection towards Evans blue dye.

2D TiO_2_ nanosheets were synthesized and integrated with rGO. The fabrication process consisted in simply grafting a titanium (Ti) precursor onto the GO nanosheets, followed by a one-pot solvothermal process.^69^ The GO nanosheets provided the template for the uniform distribution of 2D TiO_2_ nanosheets. However, compared to the pristine 2D TiO_2_ membrane, the rGO-TiO_2_ membrane had slightly lower water permeance and higher rejection towards methyl orange, possibly due to the reinforced structure and integrity. It was interesting that the developed rGO-TiO_2_ membrane demonstrated photocatalytic self-cleaning properties to degrade organic molecules.

### 3.6 Horizontal GOMs

In comparison to the approach of making substances permeate through the nanosheets in a vertical direction to the membrane, when molecules are made to permeate horizontally to the membrane, tortuous pathways can be mitigated which leads to faster transport and a precise sieving mechanism. A depiction of the flow of He, H_2_ and CO_2_ gas molecules horizontally through GO nanosheets is shown in Fig. 6d. Han *et al.* established this by utilizing heat treatment to fabricate GOMs with angstrom channels to allow horizontal transport of water, ions and gases.^70^ By tuning the sieving channel size, these horizontal membranes displayed superior permeance and selectivity for H_2_/CO_2_ and He/CO_2_ when compared to vertical GOMs (Fig. 6e). Another current challenge is the swelling of the nanosheets in liquid solutions. Typically, when GOMs are immersed in water, water molecules intercalate between the GO sheets which causes swelling. The average *d*-spacing of such membranes is 13.5 Å and their sieving properties are dependent on the relative humidity surrounding them. Nair's group described a scalable yet simple method by physical confinement to achieve interlayer *d*-spacings from ∼9.8 Å to 6.4 Å to attain graphene-based membranes with limited swelling and 97% rejection for NaCl.^71^ They investigated the interlayer spacing and ion permeation rate through these fine-tuned GO laminates at different relative humidities and found that the permeation rate for K^+^ and Na^+^ depended exponentially on the interlayer spacing while the water permeation correlated linearly with *d*-spacing. The physically confined GO membrane was achieved by stacking GO laminates in epoxy and was useful in water filtration due to reduced swelling, fast water transport and suppressed ion permeation.

## 4. Controlling the size of GO nanosheets

**Fig. 7:**
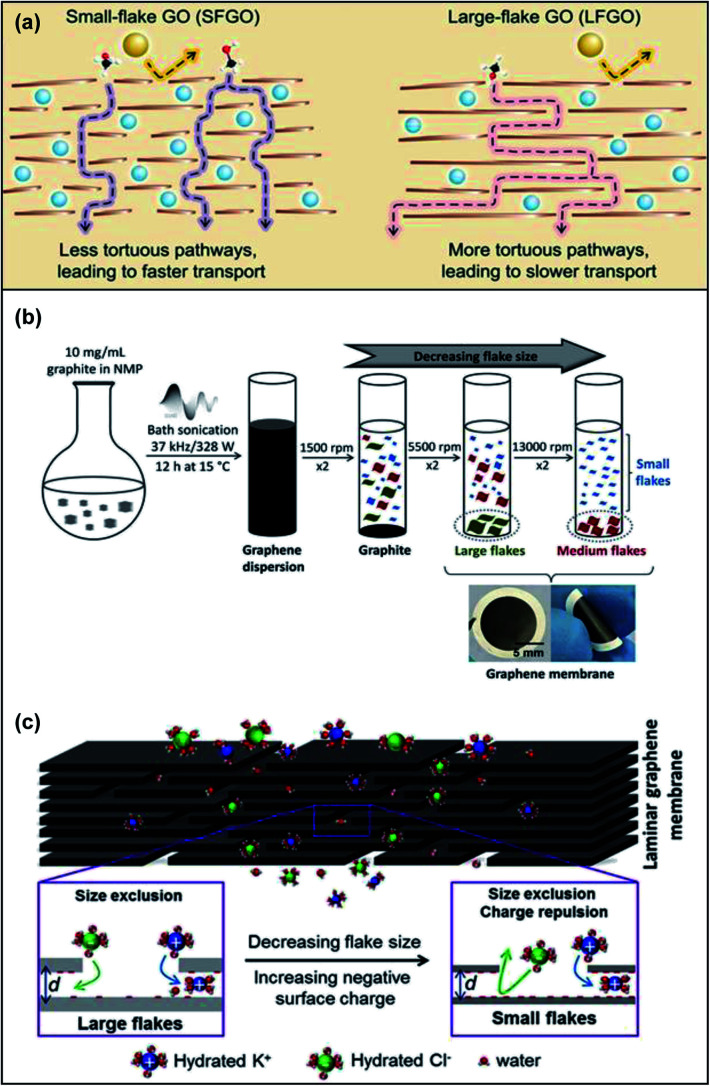
(a) Intercalation of La^3+^ (blue sphere) onto the nanosheets allowing permeation of methanol (C, black; H, white; O, red) but not other solute molecules (yellow sphere). As compared to SFGO, using LFGO makes the methanol molecule pass through a more tortuous and longer pathway, which causes lower methanol permeance. Adapted with permission from ref. 73. American Association for the Advancement of Science, copyright 2020. (b) A schematic illustration to depict the synthesis of graphene dispersions to decrease the flake size using bath sonication. (c) An illustration to demonstrate the combination of size exclusion and ionic charge repulsion utilised in SFGO as compared to LFGO when substances permeate through. Adapted with permission from ref. 74. Elsevier Science Ltd, copyright 2019.

The size and aspect ratios of GO nanosheets are important parameters which affect their physical and mechanical properties, processability and aggregation behaviour.^72^ With the goal of fine-tuning the nanochannels in GOMs by controlling the GO size, Bae and co-workers suggested a combination of two design strategies: (i) lateral dimension control and (ii) cationic crosslinking and intercalation of La^3+^ to develop an ultrathin small-flake graphene oxide (SFGO) membrane for high-performance ultrafast organic solvent nanofiltration applications in the pharmaceutical industry.^73^ A brief depiction of the shortened transport pathway of utilizing SFGO as compared to its large-flake counterpart is shown in Fig. 7a. Fast solvent permeation through a short and less tortuous pathway was achieved as the SFGO-La^3+^ membrane was observed to be >4.2-fold shorter than its large-flake counterpart after subjecting the membranes to specifically timed ultrasonication. In their comparison study between SFGO-Co^2+^ and SFGO-La^3+^, the authors concluded that only the latter was able to form sufficiently large networks to form a continuous laminate on a nylon substrate. Other techniques used to manipulate the size of GO include size-selected centrifugation (Fig. 7b). Dryfe's group used this method to prepare highly stable low oxygen content GOMs with no swelling which utilized physical sieving and charge repulsion for ion sieving in water purification applications (Fig. 7c).^74^ The mobility of ions such as chloride decreased as the lateral flake length and thickness decreased. The nanochannels formed in small flakes were observed to be more complex, which formed a highly tortuous pathway as compared to large flakes. Chiral amplification methods can also be utilized to fine-tune the size of these GOMs. Chen *et al.* discovered that the enantioseparation performance of chiral selector functionalised GOMs such as l-phenylalanine-GOMs was enhanced for membranes fabricated using small GO size due to the increased apertures and short tortuous pathway on their surfaces.^75^

## 5. Chemical modification on GO nanosheets

**Fig. 8:**
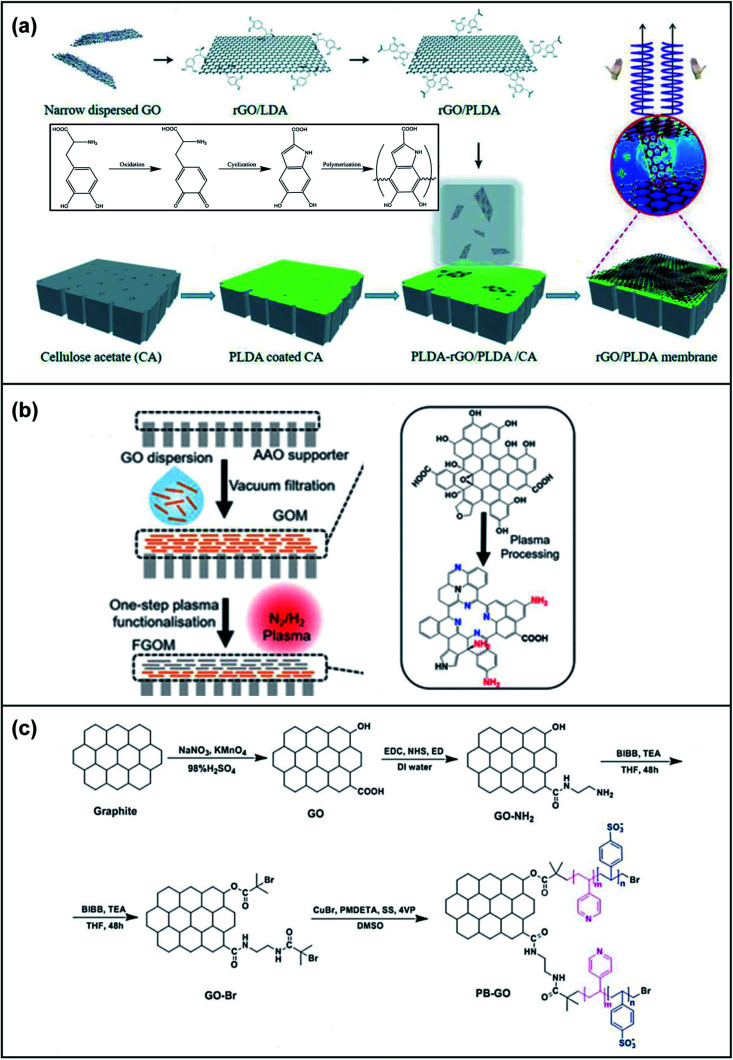
(a) An illustration of the fabrication of GO membranes using chiral amplification. Adapted with permission from ref. 78. Royal Society of Chemistry, copyright 2020. (b) Left: An illustration of the preparation steps of functionalized graphene oxide (FGO) nanosheets using a one-step plasma processing method. Right: The chemical structure of graphene oxide (GO) nanosheets and plasma FGOMs. Adapted with permission from ref. 79. American Chemical Society, copyright 2021. (c) A schematic illustrating the synthetic pathway of PB-GO. BiBB: 2-bromo-2-methylpropanoyl bromide. Adapted with permission from ref. 80. Elsevier Science Ltd. Copyright 2020.

Chemical modification techniques are effective and extensively applied to improve the membrane separation performance of GOMs.^76^ Yu's group investigated the relationship between different oxygen functional groups on GO sheets and the water purification performance of GOMs.^77^ GO nanosheets were chemically modified to construct nanofiltration membranes mostly containing carboxylic acid (–COOH), hydroxyl (–OH) and epoxy (–COC–) functional groups. The *d*-spacings of the three GOMs were 15.06 Å (–COOH), 14.27 Å (–OH) and 13.46 Å (–COC–), respectively. The difference of the GOM *d*-spacing could be attributed to the hydrophilicity and electrostatic repulsion of the oxygen-containing functional groups. The GOM with more carboxylic acid groups had stronger interaction with water molecules and the water molecules enlarge the *d*-spacing. Meanwhile, the water transport of these GOMs was primarily affected by the *d*-spacing, showing a water permeance of 5.94 (–COOH), 7.22 (–OH) and 9.19 (–COC–) L m^−2^ h^−1^ bar^−1^. In another study, Zhang *et al.* constructed chiral compound modified self-assembled GOMs with robust structural integrity in deionized water for at least 270 days. The chiral levodopa crosslinked GOMs were highly stable in acidic pH 3 and alkaline pH 10 environments, resistant to detachment when sonicated for 2 h and stable under high shear forces for cycles of 8 h without any change in appearance (Fig. 8a).^78^ Moreover, the chiral compound levodopa could decrease the interlayer spacing and create chiral sites on the GO nanosheets, resulting in high selectivity. Nitrogen functional groups such as amine groups and polarized nitrogen functional groups have also been used to manipulate the GO sheets chemically. Lei and co-workers developed an ultrathin functionalized GO membrane of 50 nm thickness with enriched nitrogen functional groups by simultaneously introducing nitrogen functional groups to enhance metal ion sieving by one-step controlled plasma processing (Fig. 8b).^79^ The selectivity of such GO-based membranes for mono/divalent cations was determined to be 10-fold higher than that of pristine GOMs. An ultrahigh water/salt selectivity of approximately 4.31 × 10^3^ was also achieved when applying K^+^, Na^+^, Ca^2+^, and Mg^2+^ ions to evaluate the permeation rates. Notably, as the duration of plasma processing was increased, the ion permeation rates decreased. A combination of strategies has also been applied to these GO frameworks to achieve advanced membrane separations. Zhang *et al.* utilized atom transfer radical polymerisation (ATRP) and crosslinking of glutaraldehyde to fabricate a low surface charged antibacterial poly(styrenesulfonic acid-*co*-4-vinylpyridine) brush-modified GO (PB-GO) nanosheet with high water permeability, low sodium sulfate rejection, excellent retention of dye molecules and high solute–solute selectivity for the fractionation of textile wastewater (Fig. 8c).^80^

## 6. Conclusions

This review provides a timely update on GO-based membranes for separations in gas, water and organic solvents. We have summarized recent publications on modulating and modifying the GO framework to improve the membrane properties and separation performance. Tuning the interlayer spacing *via* crosslinking and intercalating has been extensively explored using crosslinkers, polymers, nanoparticles, ions and 2D materials. Besides, designing the GO framework to make the transport flow permeate horizontally has also been attempted to manipulate the pathway. Controlling the size of GO nanosheets and modifying the functional groups on GO have been explored to modify the GOM and enhance its performance. All these approaches can influence the membrane performance to a certain degree, and some of them can even enhance the flux to over ten times. Thus, it is promising to tune the GO framework and enhance the separation performance of GOMs. Future studies may deploy other novel materials or modify the nanomaterials to improve their affinities with GO. Functional nanomaterials, such as photocatalysts and stimuli-responsive materials, may have potential to be incorporated with GO to fabricate smart and multifunctional GOMs. In addition, innovative studies on designing the structure of the GO framework may be promising to achieve much excellent performance. However, it should be mentioned that most current GOMs are still in the lab phase and not suitable for industrial utilization. Studies on scaling up GO-based membranes and making GOM modules may be another interesting topic to be explored.

## Conflicts of interest

There are no conflicts to declare.

## Supplementary Material
